# Evaluation of Basic Life Support First Responder Naloxone Administration Protocol Adherence

**DOI:** 10.7759/cureus.18932

**Published:** 2021-10-20

**Authors:** Joshua Mastenbrook, Daniel Emrick, Laura D Bauler, James Markman, Tyler Koedam, William Fales

**Affiliations:** 1 Emergency Medicine, Western Michigan University Homer Stryker MD School of Medicine, Kalamazoo, USA; 2 Student Affairs, Western Michigan University Homer Stryker MD School of Medicine, Kalamazoo, USA; 3 Biomedical Sciences, Western Michigan University Homer Stryker MD School of Medicine, Kalamazoo, USA; 4 General Surgery, Mount Carmel Graduate Medical Education, Grove City, USA

**Keywords:** first responder, prehospital care, basic life support, naloxone, overdose, opioid

## Abstract

Objectives: Opioid overdoses have become a significant problem across the United States resulting in respiratory depression and risk of death. Basic Life Support (BLS) first responders have had the option to treat respiratory depression using a bag-valve-mask device, however naloxone, an opioid antagonist, has been shown to quickly restore normal respiration. Since the introduction of naloxone and recent mandates across many states for BLS personnel to carry and administer naloxone, investigation into the adherence of naloxone use standing protocols is warranted.

Methods: This preliminary study examined 100 initial cases of BLS first responder administration of naloxone for appropriate indications and protocol adherence.

Results: This study found that n=22/100 naloxone administrations were inappropriate, often given to patients who were not suffering from respiratory depression (n=11/22). Positive pressure ventilation (PPV) was not administered prior to naloxone in n=56/100 cases, of which n=42/100 had an inadequate respiratory effort documented. For patients with a known history of substance use disorder, there was a significant increase in administration of naloxone prior to PPV (60%; n=33/55) compared to patients without a known history (30%; n=9/30).

Conclusion: Overall these preliminary data suggest that during BLS naloxone administration, the majority of cases did not follow at least one component of the standard protocol for patients with respiratory depression. This study suggests that further education and more research are needed to better understand the decision-making processes of prehospital providers to ensure adherence to standard protocols.

## Introduction

Background

Throughout the United States, opioid use disorder and overdoses have been a significant problem facing emergency medical responders, dramatically increasing over the last two decades. According to the Centers for Disease Control, from 1999-2017 more than 400,000 people died as the direct result of an overdose involving opioids [1]. In 2014, deaths due to opioid overdoses (n=47,055) totaled 1.5 times those due to motor vehicle crashes, with 61% of these overdose deaths being linked to illicit and non-prescribed opioids [2]. 

Importance

Naloxone has been shown to be effectively employed in an emergency setting to reverse the effects of an opioid overdose [3-5]. However, studies have focused on the use of naloxone by paramedics rather than basic life support (BLS) providers, likely because naloxone administration had not been incorporated into the National EMS Scope of Practice Model as a BLS provider skill (until 2017), and nationally, states have traditionally limited this intervention to paramedics and advanced emergency medical technicians [6-9]. In 2013, only 12 states allowed emergency medical technicians to administer naloxone, and only three states (Maryland, New York, Ohio) allowed emergency medical responders to administer naloxone [8]. Nevertheless, the rapid positive outcomes seen from naloxone administration studies have augmented the support for expanding access of naloxone to law enforcement officers and basic level prehospital providers, who are often the first to arrive on scene [6,10]. BLS providers typically utilize a positive pressure bag-valve-mask device in combination with an airway adjunct to effectively treat respiratory depression independent of its causative factors until more advanced providers arrive on scene. Respiratory depression and arrest are features of many medical conditions, such as congestive heart failure, chronic obstructive pulmonary disease, asthma, seizures, intracranial hemorrhage, and head trauma. Relative to this paper, toxicities resulting from opioids, alcohols, barbiturates, and benzodiazepines can cause respiratory depression and arrest, The additional expertise and resources available to paramedics enables them to differentiate and treat more etiologies of respiratory depression than a BLS provider.

Goals of this investigation

In response to the rising mortality of the opioid epidemic across the nation, numerous states have established legislation to allow for naloxone administration by BLS providers and lay responders. Michigan became the 26th state to make naloxone available to BLS agencies, in Public Act 312 House Bill 5404 on October 14, 2014, but only the third state to mandate that those agencies carry the medication on their licensed vehicles [11]. Proponents of this mandate believed that increased naloxone availability would reduce the mortality currently associated with the opioid epidemic. Critics expressed concerns with cost and training, as well as the significant concern that naloxone may be administered in lieu of other indicated interventions such as bag-valve-mask ventilation [12]. As research into the validity of this latter concern is currently lacking, the state mandate provides a unique opportunity to investigate whether BLS first responder use of naloxone will affect the utilization of standard respiratory depression treatment. The primary outcome of this study is to determine the rate of adherence to the standard protocol for naloxone administration by BLS first responders.

## Materials and methods

Study design and selection of participants

A preliminary retrospective chart review was performed on the Michigan Emergency Medical Services Information System (MI-EMSIS) database, a mandatory reporting database. MI-EMSIS contains patient care report information from all licensed emergency medical service agencies in Michigan. The data were queried based on a filter for medication administration of "naloxone" or "narcan." Data were downloaded into a spreadsheet in sequential order of record creation in the database. Inclusion criteria included administration of naloxone by BLS trained personnel prior to the arrival of advanced life support (ALS), regardless of outcome or indication, to an adult patient (greater than 18 years of age). Cases that lacked data to determine use of airway interventions (such as supplemental oxygen, nasal cannula, non-rebreather, bag-valve-mask or airway adjunct use such as a nasal trumpet or oropharyngeal airway), pre/post naloxone mental status (as assessed by Glasgow Coma Scale or Alert, Voice, Pain, Unresponsive (AVPU) categorization) and respiratory status (based on a documented respiratory rate or description provided in the narrative) were excluded. Each case (spreadsheet row) was reviewed sequentially until accruing one hundred cases that met the inclusion criteria, did not include exclusion criteria, and had sufficient documentation to allow for analysis. Data were collected between October 15, 2015 and March 13, 2016.

Data were abstracted from each case, including agency licensure level, initial impression of mental status and respiratory status (Table 1), vital signs, airway interventions performed, known history of opioid abuse, timing of ALS arrival (greater than or less than five minutes in relationship to when the BLS provider was prepared to administer naloxone) and the case narrative. A response to naloxone was defined as an improvement in respiratory rate or an improvement in mental status. Due to the varied and often incomplete documentation, a broader outcome for improvement in response to naloxone than just documentation of post-naloxone respiratory rate was used. Cases were reviewed for inclusion by three independent investigators (JOM - medical student, TJK - emergency medicine resident, JDM - EMS physician). BLS personnel were defined as those responding as part of an organized EMS-licensed non-transport non-ambulance first response agency. The first response agencies in Michigan are referred to as “Medical First Responders” in lieu of the National Registry term, “Emergency Medical Responder.” This study was determined to be exempt following review by the Michigan Department of Health and Human Services’ Institutional Review Board.

**Table 1:** Initial Clinical Impression of Patient upon BLS First Responder Assessment Each patient record was independently evaluated by two clinicians (JOM/TJK) and the clinical impression of the patient was assigned to a single category based upon the presenting signs and symptoms.  In cases where the two independent scores differed, a third clinical opinion (JDM) was used as a tie breaker following discussion among the three researchers. Abbreviations used: BLS (Basic Life Support), AED (Automated External Defibrillator), GCS (Glasgow Coma Scale), AVPU (Alert, Voice, Pain, Unresponsive)

1	Cardiac Arrest	Lack of palpable pulse by BLS first responder or continuation of bystander-initiated compressions after BLS arrival, or Statement of cardiac arrest in narrative, or AED use with shock delivered.
2	Respiratory Arrest	Documented respiratory rate of 0, or Narrative documentation indicating complete lack of respiratory effort
3	Respiratory Failure	Respiratory rate of <8, or BLS documentation of agonal respirations, or Narrative report implying significantly impaired breathing
4	Unconscious w/ altered respiration	GCS <8, or AVPU of P or U described in narrative, or BLS statement of unconscious or unresponsive AND cyanosis, hypoxia or mildly impaired respiration
4.5	Unconscious w/ unknown respiration	GCS <8, or AVPU of P or U described in narrative, or BLS statement of unconscious or unresponsive AND no documentation of respiration rate
5	Unconscious w/ adequate respiration	GCS <8, or AVPU of P or U described in narrative, or BLS statement of unconscious or unresponsive AND RR >8 with no documentation of cyanosis or hypoxia
6	Altered LOC with adequate respiration	GCS 8-13, or AVPU of V described in narrative, or BLS statement of altered mentation AND RR >8 with no documentation of cyanosis or hypoxia
7	Conscious w/ adequate respiration	GCS 14 or similar narrative description AND RR >8 with no documentation of cyanosis or hypoxia
8	Alert & Oriented	GCS 15, or AVPU of A described in narrative AND RR >8 with no documentation of cyanosis or hypoxia
9	Indeterminate	Unable to determine patient status based on documentation after physician collaboration

Analysis

**Figure 1:**
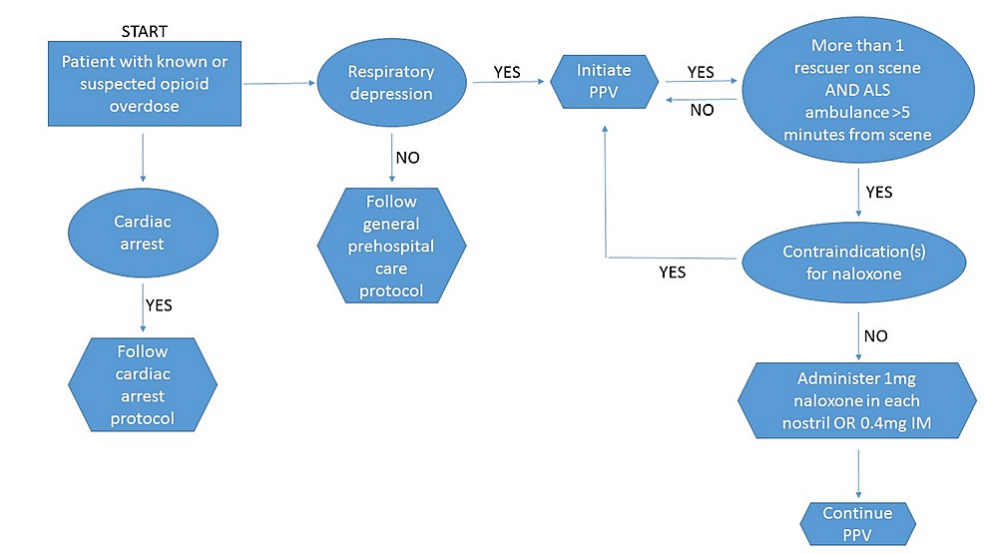
Michigan Naloxone Administration Protocol (v06042015) Flow diagram of the Michigan Naloxone Administration Protocol. Note the early emphasis on PPV along with adequate scene safety with more than 1 rescuer on scene and ALS >5 minutes from the scene prior to naloxone administration by BLS first responders. Contraindications for intranasal naloxone stated in the protocol are: nasal trauma, epistaxis, nasal congestion/significant discharge, and known cocaine use. The stated treatment goal is adequate patient breathing effort and not necessarily an awakened patient. Abbreviations: PPV = positive pressure ventilation; ALS = advanced life support; BLS = basic life support.

The initial patient clinical presentation upon BLS first responder assessment was independently categorized based on the respiratory rate and narrative descriptions of each case by two investigators (JOM, TJK) based on the categories defined in Table 1; in cases where numerical designations differed, a third reviewer (JDM) weighed in and consensus was reached by discussion. To limit bias, data were abstracted and scored by JOM/TJK, individuals that were not directly involved in designing the study. Categories two to four (Table 1) were determined to represent appropriate patients for consideration of naloxone administration based upon the State of Michigan EMS protocols (Figure 1). Descriptive statistics were calculated using Microsoft Excel. Chi-square analysis was conducted to compare use of ventilation prior to naloxone administration and knowledge of substance use disorder history, with an alpha of p < 0.05.

## Results

Characteristics of study subjects

Querying of the MI-EMSIS database for naloxone or narcan administration between incident dates of October 15, 2015 and March 13, 2016 yielded 478 records. The date range did not need to be extended further than March 13, 2016 as the objective of obtaining 100 cases of BLS administered naloxone was met. The majority of excluded cases were due to non-BLS administrations of naloxone. Of the included 100 BLS first responder naloxone administrations, 24% of patients were female, 58% of patients were male, and 18% were unclassified in the EMS record with an average age of 37 (range: 18 to 89 based on documentation of age in 52/100 records). Identification of the specific opioid is difficult in the field, but patient or bystander reports, or paraphernalia, indicated a specific agent in 47% of confirmed reversals. Of all cases where the agent was identified (n=47/100), heroin was reported in 70.2% (n=33/47) of the overdoses, followed by methadone in five cases (10.6%), oxycodone, hydrocodone and tramadol each in two cases (4.3%), and fentanyl and morphine each in a single case (2.1%).

Main results

**Figure 2:**
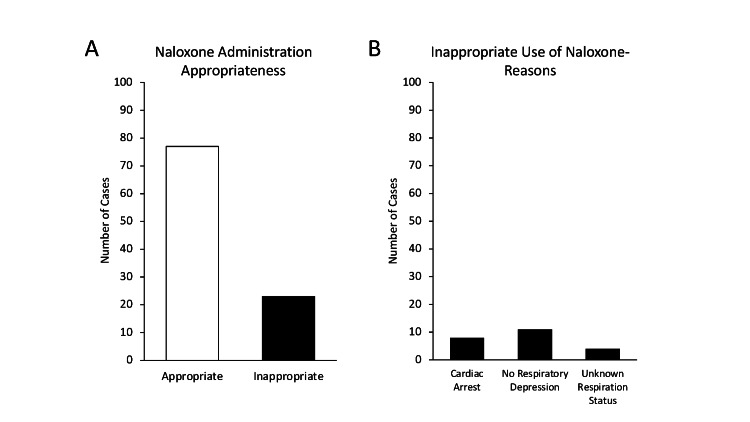
Appropriateness of Naloxone Administration by Basic Life Support Providers A) Appropriateness of naloxone administration was determined based upon patient level of consciousness, respiratory effort, and detection of cardiac arrest. B) For inappropriate naloxone administration, the number of cases with each reason for inappropriate use was determined.

To examine for appropriateness of the administration of naloxone, each administration was evaluated for respiratory effort, patient level of consciousness, and presence of cardiac arrest. The majority, 77% (n=77/100) of naloxone administrations were provided to individuals for whom the intervention was indicated based on documentation of inadequate respirations with a pulse. Thus, the remaining 23% of naloxone administrations were deemed inappropriate by the study according to state protocol, either due to cardiac arrest in the absence of opioid suspicion or adequate respiratory rate, although 4/100 cases did not contain enough documented information to abstract whether or not the patient had adequate respirations - these were included in the inappropriate naloxone administration set (Figure 2). Of the inappropriately administered doses, 35% (n=8/23) were given during cardiac arrest resuscitation by a BLS first responder. The remaining 48% (n=11/23) of inappropriate administrations were given to patients not experiencing respiratory depression, and 17% (n=4/23) did not contain enough documentation to abstract adequacy of respirations prior to administration of naloxone; however, enough data were present to exclude cardiac arrest.

**Figure 3:**
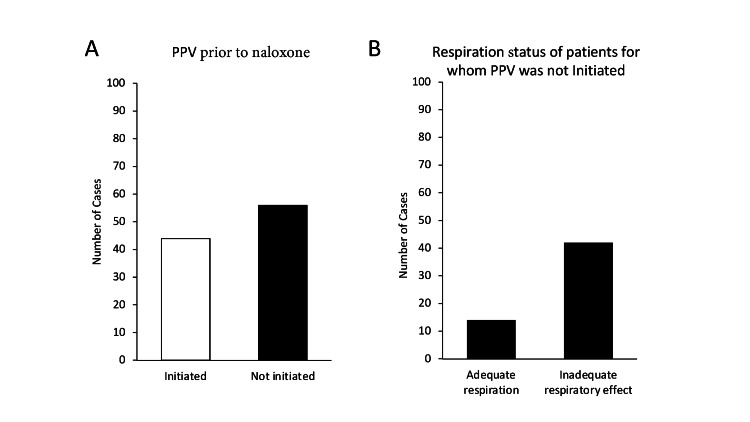
Positive Pressure Ventilation Administration to Patients A) Number of cases where PPV was or was not initiated prior to naloxone administration. B) Respiratory status for patients for whom PPV was not initiated prior to administration of naloxone.

State EMS protocol requires that positive-pressure ventilation (PPV) be attempted prior to naloxone administration and continued until the patient resumes adequate spontaneous breathing. The compiled data reveal that PPV was not initiated in 56% (n=56/100) of patients prior to naloxone administration. Of these 56 patients, 42 (75%), had documentation supporting an inadequate respiratory effort, thus needing ventilatory support (Figure 3). Additionally, one of the eight cardiac arrest patients who received naloxone during resuscitative efforts did not receive any PPV prior to naloxone administration. 

**Figure 4:**
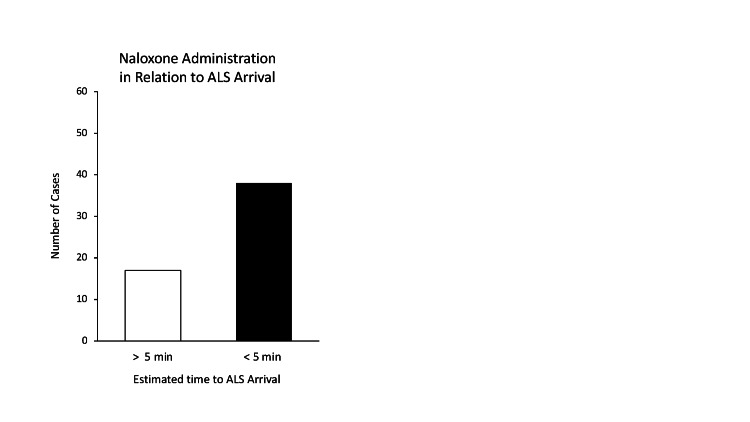
The Timing of Naloxone Administration with Regard to Estimated Time of Advanced Life Support (ALS) Arrival For cases where ALS arrival times were indicated in the chart, appropriateness of naloxone administration was determined based on the ruling that naloxone should not be administered when ALS personnel are estimated to arrive within five minutes.

The Michigan EMS naloxone administration protocol permits that naloxone be given by BLS first responders only after confirmation that an ALS ambulance is greater than five minutes from the scene. Of the 55 events documenting ALS proximity to the scene, 69% (n=38/55) administered naloxone within the five-minute ALS arrival window (Figure 4). The statewide prehospital protocol was revised from this 2015 version in 2017 and no longer has the requirement that the ALS ambulance be greater than five minutes out from the scene. The updated protocol continues to require that positive pressure ventilations be established and more than one rescuer be on-scene prior to naloxone administration.

Secondary results

**Figure 5:**
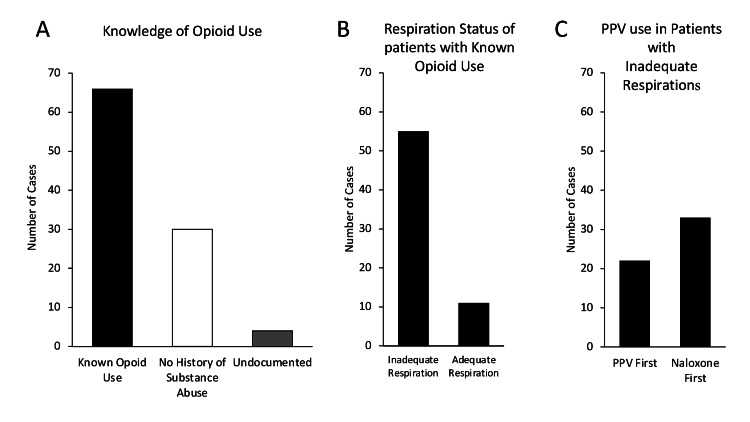
Administration of Naloxone Based upon the Providers' Knowledge of Opioid Use A) Number of cases where opioid use was known by providers. B) The respiratory status for patients who received naloxone who had a known use of opioids. C) Of patients with known opioid use, the order of care (naloxone or positive pressure ventilation first) for patients with inadequate respirations was determined.

Opioid use disorder history was known by BLS first responders in 66% (n=66/100) of patients, either by personal knowledge, anecdote, or via the dispatcher. Among these 66 cases, 55 patients had documented respiratory inadequacy. PPV was attempted prior to naloxone intervention in 40% (n=22/55) of these patients. In comparison, patients without a known substance use disorder history, found with inadequate respirations by BLS first responders, received PPV prior to naloxone 70% (n=21/30) of the time (Figure 5). When analyzing the association between BLS first responder knowledge of substance use disorder history and the tendency for PPV prior to naloxone administration among patients found and documented to have inadequate respirations, a chi-square value of 6.989 (p = 0.0082) resulted, suggesting BLS first responders are more likely to administer naloxone prior to PPV if they know the patient has a history of substance use disorder.

## Discussion

Current literature has demonstrated the efficacy of BLS first responder administration of naloxone, showing positive outcomes for opioid overdose patients with respiratory depression [13-18]. However, the appropriateness of naloxone delivery in accordance with current BLS protocols has not been extensively examined. This study evaluated BLS first responder behaviors in the context of airway management protocols following a statewide naloxone mandate for treating potential opioid overdose patients. Prior to this mandate, Michigan BLS first responders (non-ambulance, non-transport) did not carry naloxone.

While this study is preliminary, analysis of these 100 administrations of naloxone by BLS first responders, following the Michigan Public Act 312 (2014) implementation date of October 15, 2015, demonstrates there are a number of pitfalls to this approach of addressing a public health crisis, most notably the lack of adherence to established treatment protocols designed to ensure the delivery of high-quality standardized care. A number of factors may have contributed to this lack of adherence, including insufficient training, individual provider bias, or the “new tool in the toolbox” (novelty) phenomenon. In addition to education, altering protocol(s) to include duration of time for PPV before naloxone administration, might be helpful. 

The statewide BLS first responder naloxone administration protocol directs rescuers to ensure PPVs are delivered prior to considering naloxone administration. Only 44% of the 100 naloxone administration patients received PPV by BLS first responders prior to the antidote. As an effective first-line treatment for all causes of hypoventilation or respiratory arrest, failure to quickly initiate PPV has the potential to adversely affect patient outcomes, especially if opioid use is complicated by medical comorbidities or other toxicities. PPV has the benefit of delivering high concentrations of oxygen to correct hypoxia, ventilating to correct hypercarbia, and theoretically preventing adrenaline surge-induced pulmonary edema following reversal of an apneic patient [19].

If one examines only those patients that truly needed PPV, based on documentation of absent or inadequate respirations (85/100 patients), only 51% received PPV prior to naloxone administration. A systematic review examining adherence to protocols during prehospital care suggests that adherence widely varies across studies, and may not even be tracked [20]. Additionally, it has been suggested that provider experience, awareness and preference of protocol, as well as patient-specific and organization factors contribute to providers deviating from protocol [20,21]. Given the current literature, findings of low adherence to the naloxone protocol are not surprising. 

Following the implementation of the BLS first responder naloxone law, each of the 60 different EMS oversight authorities within the state was tasked with educating its credentialed prehospital providers on the new naloxone administration protocol. Across agencies, there was likely an array of training curricula to educate BLS first responders about the new naloxone protocol. The learning modality, in addition to personal and systemic factors, has been shown to impact retention of knowledge and performance of procedures by prehospital providers [22-25]. While the extent to which these factors impact BLS providers' adherence to protocol was not specifically examined in this study, these findings suggest that training differences could have contributed to the rates of improper naloxone use, as has been seen in other studies [26,27]. Nearly one-quarter of patients in this study received naloxone prior to the standard protocol for respiratory depression management, additionally several patients in cardiac arrest (n=8/100) received naloxone which accounted for 35% of the inappropriate naloxone administrations. All of the reported cardiac arrest victims received chest compressions, but only seven of the eight cardiac arrest patients received PPV prior to naloxone administration. The American Heart Association does allow for the caveat of considering naloxone administration to a presumed cardiac arrest victim when an opioid overdose is also suspected with the rationale that the patient may be in pseudo-arrest, potentially apneic and significantly bradycardic to the point that a 10-second pulse check may miss manual palpation of a pulse [28]. Nonetheless, professional rescuers should focus on delivering high-quality respiration prior to considering naloxone administration. 

One unique concept tested in this study was the idea that there could be provider bias towards providing naloxone to patients who had a known association with substances, based on previous encounters, dispatch information, or family member reporting, and this knowledge could affect decision making on airway and breathing management. The data suggest a significant correlation exists between BLS first responder knowledge of opioid use and disregard of standard airway management protocols in relation to the naloxone administration protocol. Patients who BLS first responders knew had a history of substance use disorder were more likely to receive a dose of naloxone prior to PPV. BLS first responders may overlook appropriate airway intervention and instead first use naloxone, in contradiction of current state protocol. A counter-argument could be made that the naloxone and PPV were administered simultaneously, however due to having to extract data from the narrative when time values were not documented, the order of treatment had to be inferred based on the order of documentation. Thus, PPV may not have truly been delayed despite what the documentation may suggest. No (non-cardiac arrest) patients were reported to have died on-scene as a result of providing naloxone prior to PPV, however, not all patients responded positively to naloxone, likely due to non-opioid etiology (i.e. hypoglycemia) or concomitant use of centrally acting depressants. The data were heavily weighted toward naloxone administration in young adult males. This might suggest additional provider bias in the population of patients receiving naloxone. 

The novelty concept along with a desire to best treat patients is supported by the observation that a significant number of BLS first responders administered naloxone despite knowledge that the ALS ambulance was within five minutes of the scene. State protocol required that if the ALS ambulance was within five minutes of the scene, BLS first responders should manage the hypo-ventilating patient with PPV instead of administering naloxone. With this new tool available for use, knowledge of the effectiveness of the antidote, a high-stress situation, and a genuine interest in helping the suspected opioid overdose patient improve, it is not surprising that naloxone was being administered even with short ambulance response times in a significant number of cases. 

Limitations

As a preliminary hypothesis-generating study, this study is not without limitations. All data were derived from the Michigan EMS Information System - an online database that contains prehospital patient care report data; it is not a registry. Therefore, there may have been relevant data that were omitted or entered in error. Additionally, the on-scene patient acuity may result in recall bias during documentation. However, this database is the only source of data currently available for a rapid assessment of protocol adherence across Michigan EMS agencies. Additionally, this was a relatively small study, evaluating only 100 cases of naloxone administration by BLS first responders post mandate. Further evaluation using a larger sample size may be beneficial for better characterization of prehospital trends in suspected opioid overdose case management. It is possible that the results in this study may not be globally applicable or representative of all states. The data contained records from 21 counties across Michigan, ranging from between one and 16 records per county. There is also limited information regarding the methods utilized to educate providers across such a wide variety of sites. To limit bias, data were abstracted and scored by individuals that were not directly involved in designing the study (TJK, JOM). Nonetheless, they were familiar with the study design and authors of the study and therefore there is the potential for investigator selection bias. Finally, this study only examined protocol adherence in 100 cases of BLS naloxone administration and did not determine a baseline adherence rate for airway intervention in this population prior to the introduction of naloxone as a tool readily available to BLS first responders.

## Conclusions

As illicit opioid usage rates continue to rise, tools such as naloxone have become more widely available to reverse the mortality associated with opioid overdoses. Calls to action can be heard across the county, often manifesting in legislative efforts such as Michigan’s BLS first responder naloxone mandate. Overall, our hypothesis-generating study suggests that during these 100 cases of BLS naloxone administration, the majority of cases did not follow at least one component of the standard protocol for patients with respiratory depression. While most naloxone administration events were indicated based on clinical presentation, more than half violated the state EMS protocol and were administered without preceding oxygenation and ventilatory support and/or within five minutes of ALS arrival. In addition, we noted the tendency of first responder bias to administer naloxone prior to initiating PPV in individuals known to the responders to have a history of substance use disorders. This small study showed significant protocol lapses that warrant larger-scale investigation to determine if a lack of PPV with naloxone use is widespread.
